# Cardiovascular safety of calcium, magnesium and strontium: what does the evidence say?

**DOI:** 10.1007/s40520-021-01799-x

**Published:** 2021-02-09

**Authors:** Elizabeth M. Curtis, Cyrus Cooper, Nicholas C. Harvey

**Affiliations:** 1 MRC Lifecourse Epidemiology Unit, University of Southampton, Southampton General Hospital, Southampton, SO16 6YD UK; 2 Rheumatology Department, University Hospital Southampton NHS Foundation Trust, Southampton, UK; 3 NIHR Oxford Biomedical Research Centre, University of Oxford, Oxford, UK; 4 NIHR Southampton Biomedical Research Centre, University of Southampton and University Hospital Southampton NHS Foundation Trust, Southampton, UK

**Keywords:** Osteoporosis, Epidemiology, Calcium, Magnesium, Strontium, Cardiovascular

## Abstract

Calcium, magnesium and strontium have all been implicated in both musculoskeletal and cardiovascular health and disease. However, despite these three elements being closely chemically related, there is marked heterogeneity of their characteristics in relation to cardiovascular outcomes. In this narrative review, we describe the relevant evidential landscape, focusing on clinical trials where possible and incorporating findings from observational and causal analyses, to discern the relative roles of these elements in musculoskeletal and cardiovascular health. We conclude that calcium supplementation (for bone health) is most appropriately used in combination with vitamin D supplementation and targeted to those who are deficient in these nutrients, or in combination with antiosteoporosis medications. Whilst calcium supplementation is associated with gastrointestinal side effects and a small increased risk of renal stones, purported links with cardiovascular outcomes remain unconvincing. In normal physiology, no mechanism for an association has been elucidated and other considerations such as dose response and temporal relationships do not support a causal relationship. There is little evidence to support routine magnesium supplementation for musculoskeletal outcomes; greater dietary intake and serum concentrations appear protective against cardiovascular events. Strontium ranelate, which is now available again as a generic medication, has clear anti-fracture efficacy but is associated with an increased risk of thromboembolic disease. Whilst a signal for increased risk of myocardial infarction has been detected in some studies, this is not supported by wider analyses. Strontium ranelate, under its current licence, thus provides a useful therapeutic option for severe osteoporosis in those who do not have cardiovascular risk factors.

## Introduction

That the cardiovascular safety of calcium, magnesium and strontium is under consideration might indicate concerns across this particular section of the periodic table. The reality is that the cardiovascular associations of these three elements are markedly heterogeneous, as is their use in medicine. Calcium is perhaps the most studied, with evidence for modest efficacy in terms of fracture risk reduction when taken with vitamin D supplementation [1]; evidence over the last 5 years has suggested associations between calcium or calcium/vitamin D supplementation and increased cardiovascular risk [1]. However, the links are far from conclusive, with other studies demonstrating no association; key considerations such as a potential mechanism remain to be delineated [1]. Magnesium has little evidential support in terms of fracture prevention, although is often sold as a component of bone health nutritional supplements [2, 3]; greater dietary intake and serum concentrations appear to be protective for cardiovascular outcomes [4–7]. Strontium has been used in the form of strontium ranelate as a therapy for osteoporosis [8]. Whilst it has efficacy for vertebral and nonvertebral fractures, initial trials demonstrated an increased risk of thromboembolic disease [9, 10]. In post-marketing data, a signal for increased risk of myocardial infarction led the European Medicines Agency to change to its indication [11], and the drug was subsequently withdrawn from the market by the manufacturer, only to reappear as a generic more recently. In this narrative review, we explore this heterogeneous landscape, focusing principally on the cardiovascular safety of calcium supplementation, together with newer evidence around potential mechanisms, and appraise the evidence for links between magnesium salts or strontium ranelate and cardiovascular disease.

## Calcium

### Background

Whilst dietary calcium intake and endogenous vitamin D synthesis are sufficient for most individuals in many populations, there is evidence that supplemental approaches [12–16], particularly targeted to individuals with inadequate calcium and vitamin D status, may benefit bone mass and reduce fracture risk. In recent years, the role of calcium, together with that of concomitant vitamin D supplementation, has come under close scrutiny as a result of studies suggesting potential adverse cardiovascular effects from calcium or calcium and vitamin D supplementation [1].

### Calcium, bone health and fracture risk

Although several studies, at least in the short term, have indicated positive effects of calcium supplementation on bone mineral density [17–20], the key outcome in terms of effectiveness is fracture reduction. There have been many randomised controlled trials of either calcium alone or calcium in combination with vitamin D for fracture reduction and several subsequent meta-analyses seeking to elucidate the overall effect of this intervention [21–25]. Furthermore, the vast majority of antiosteoporosis treatments have been licensed in the context of calcium and vitamin D repletion, most usually achieved with supplementation [26]. Taken as a whole the evidence base thus supports the use of calcium in combination with vitamin D supplementation rather than as the sole agent for reduction of fracture risk, but with the magnitude of effect being modest. However, efficacy has not been demonstrated for all individual fracture types, or for calcium supplementation alone. Intervention is probably best directed, therefore, at those judged to be at high risk of calcium/ vitamin D deficiency. How this high-risk population may be defined is much debated, and the reader is referred to the guidance from the US Institute of Medicine [27]. The role of routine calcium and vitamin D supplementation as a population health strategy for fracture prevention is not robustly supported.

### Calcium supplementation, renal stones and gastrointestinal side effects

Until the BMJ publication by Bolland et al. in 2008 [28], the only potential adverse effects associated with calcium and vitamin D supplementation had been an increased risk of renal calculi and gastrointestinal symptoms. Indeed, a 2014 Cochrane review has confirmed the modest increase in renal stone risk [29], which is mainly informed by data from the Women’s Health Initiative, demonstrating that the intervention was associated with a 17% increased risk of renal stones (HR 95% CI: 1.02, 1.34). It is important to note the magnitude of this outcome in the context of the WHI study, given that there was no statistically significant decrease in hip or other fractures. The WHI investigators also examined the risk of renal stones, stratified by use of personal calcium and vitamin D supplements at baseline and adherence to study medication [30]. In the subset who did not use personal supplements, the hazard ratio for renal stones with calcium and vitamin D supplementation was 1.08 (95% CI: 0.88, 1.32); in the subset who did use personal supplements, the hazard ratio was 1.23 (95% CI: 1.01, 1.48), although the interaction term was not statistically significant. Within the personal supplements group, there was no difference in the hazard ratio for renal stones by adherence.

Gastrointestinal side effects have been relatively commonly noted in trials of calcium supplementation. Symptoms include constipation, excessive abdominal cramping, bloating, and, importantly, upper GI symptoms. Lewis et al. [31] reviewed the risk of GI side effects across seven studies included in the Bolland et al. 2010 meta-analysis [32], and in which myocardial infarction was self-reported. Overall, the risk of GI side effects was increased by 43% in the calcium/calcium and vitamin D groups [RR: 1.43 (95% CI: 1.28, 1.59); *p* < 0.001]. The authors did not identify any effect of the formulation or dose of the calcium supplement, but did find evidence of both upper and lower GI events being increased. Using the adjudicated hospital admissions for GI complaints derived from hospital discharge summaries in one Australian study, 6.8% of calcium-treated patients experienced a GI complaint (*n* = 50) compared with 3.6% (*n* = 26) allocated to placebo [RR: 1.92 (95% CI: 1.21, 3.05); *p* = 0.006]. Thus it is clear that gastrointestinal side effects are an important consideration in any strategy predicated on widespread use of calcium supplements. Importantly, the authors hypothesised that self-reported myocardial infarction may, in some cases, represent misclassified gastrointestinal events, an effect demonstrable in the calcium only trials of Bolland et al. [28], and Prince et al. [33].

### Potential cardiovascular effects of calcium supplementation: initial evidence

In their 2008 BMJ paper Bolland et al. reported the adverse event follow-up from a New Zealand randomised controlled trial of calcium supplementation (without vitamin D) [28]. Amongst 1471 postmenopausal women with a mean age of 74 years, who had been randomised to either 1 g of elemental calcium citrate or placebo, incident cardiovascular events were self-reported, and adjudicated by review of medical records. The authors analysed the events in terms of simple self-report, adjudicated self-report, and then adjudicated events with additional (non-participant reported) events from Health Registry data. A further regression analysis adjusted for covariates. A more detailed examination of the study is presented in [1]. The multiplicity of endpoints provided heterogeneous results [28], and it was notable that the baseline characteristics of the participants appeared to tend towards greater cardiovascular risk in the treatment than placebo group. The message of increased cardiovascular risk from calcium supplementation that was widely taken from this paper rests very much on the analysis of self-reported events, which were not the primary outcomes of the study. Indeed the findings were not supported by the further analysis reported in the manuscript, points which seem to be have been largely ignored in its interpretation.

### Cardiovascular outcomes in meta-analyses of calcium/vitamin D supplementation

**Fig. 1:**
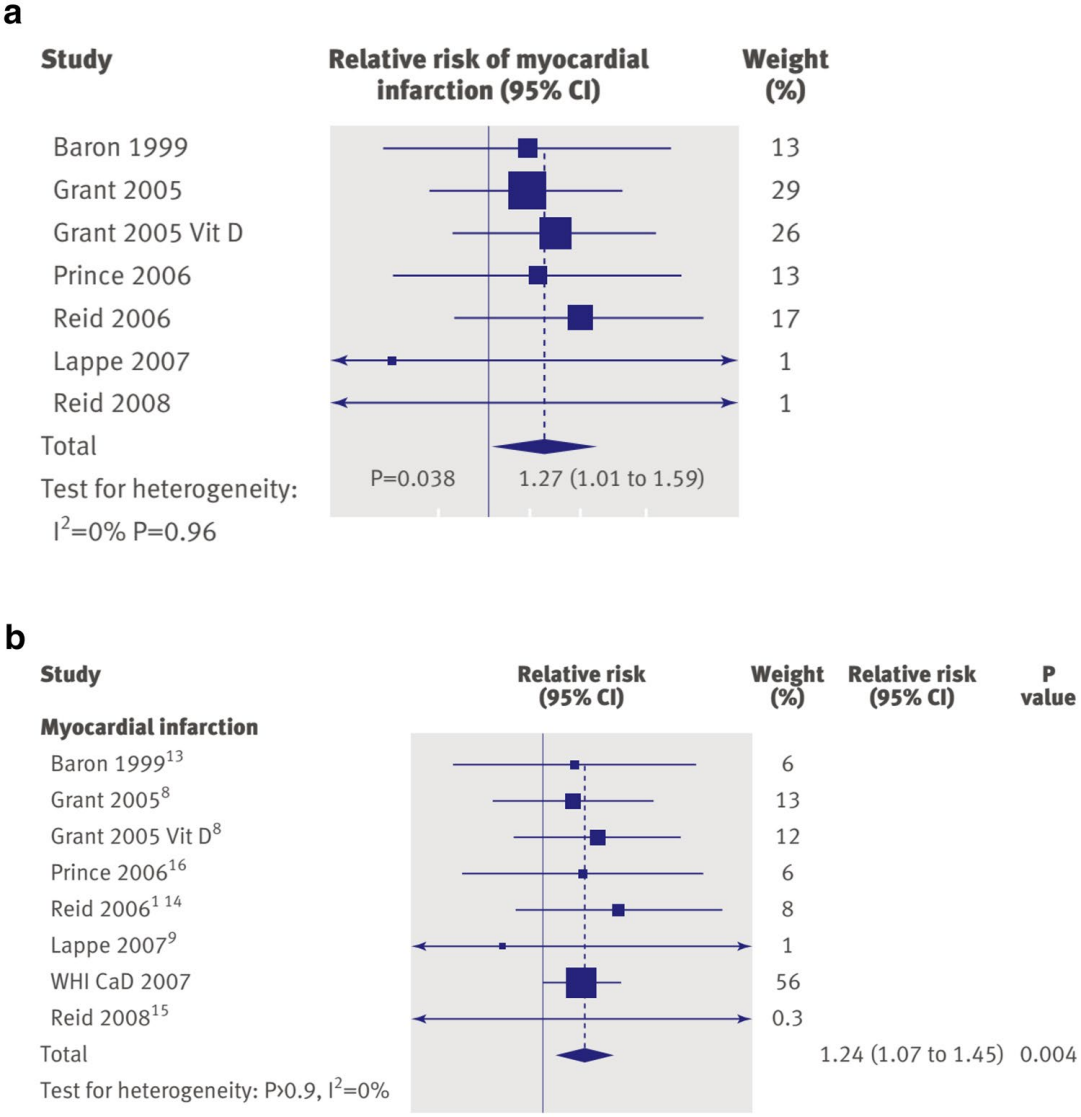
**a** Calcium supplementation and risk of myocardial infarction, with outcomes from self-report and/or verified sources. Reproduced with permission from Bolland et al., 2010 [32]. **b** Calcium and vitamin D supplementation and risk of myocardial infarction, with the outcome ascertained from self-report and/or verified sources. Reproduced with permission from Bolland et al., 2011 [121]

This initial paper was followed by a meta-analysis of randomised trials of calcium supplementation [32] combining 8151 persons in a patient level analysis, in which the RECORD study provided two-thirds of the cases and two-thirds of the myocardial infarction events (Fig. 1a). Second, the authors undertook a trial level analysis of 11,921 participants, the RECORD study providing 44% of the cases and 55% of the myocardial infarction events. In the patient level analysis, there was weak evidence of an increased risk of myocardial infarction [HR: 1.31 (95% CI: 1.02, 1.67); *p* = 0.035] but not of stroke or death, or of the composite outcome including all three [HR: 1.18 (95% CI: 1.00, 1.39); *p* = 0.057]. There was evidence of an interaction between treatment and baseline dietary calcium intake, which was only observed for the outcome of myocardial infarction. Thus the treatment myocardial infarction association appeared to be stronger above than below the median calcium intake of 825 mg per day, but there was no similar interaction for the other outcomes; there was no consistent increase in the hazard ratio for myocardial infarction associated with calcium supplementation by fifths of dietary calcium intake. In the trial level analysis, differences were similar but even smaller [myocardial infarction, HR: 1.27 (95% CI: 1.01, 1.59); *p* = 0.038; and composite end point, HR: 1.12 (95% CI: 0.97, 1.30); *p* = 0.13]. Although all events were adjudicated blind by the investigators, the included studies recorded cardiovascular outcomes in different ways with some using self-report (including RECORD), some hospital records and some death certificates.

The suggestion of an interaction between treatment and baseline calcium intake is intriguing given findings from a re-analysis of the WHI study, undertaken by the same group, Bolland et al., in 2011. They also included the WHI study in their second meta-analysis, investigating both calcium and vitamin D supplementation [34]. In this paper the authors used the WHI calcium and vitamin D study public access dataset to investigate the effect of calcium and vitamin D supplementation versus placebo in 36,282 community dwelling postmenopausal women. The WHI allowed women to take personal calcium and vitamin D supplementation in addition to the study medication, and the authors reasonably hypothesised that this might modify the effect of the trial medication. They, therefore, stratified their analysis by personal calcium and vitamin D supplementation and found that there was evidence of an interaction between personal use of supplements and allocation to calcium and vitamin D or placebo for cardiovascular events. However, the importance of this interaction is uncertain, given the lack of any clear associations in either stratum, and no real evidence of a differential pattern of relationships. There was weak evidence of an association between supplementation and clinical myocardial infarction or re-vascularisation in the group who did not use personal calcium supplementation, but there was no difference in death from all causes. Amongst those who were using personal calcium supplementation, death from all causes was actually greater in the placebo group, and both stroke and the composite outcome of clinical myocardial infarction or stroke appeared, if anything, less frequent in those allocated to calcium and vitamin D who were taking personal supplements [calcium and vitamin D group 4.8% versus placebo 5.5%, HR 0.88 (95% CI: 0.76, 1.02); *p* = 0.006]. Critically, in terms of the requirements to establish a casual relationship, there was no evidence of a dose effect according to personal calcium supplementation ranging from 0 to above 1000 mg per day.

The addition of the WHI data into a trial level meta-analysis with two other studies of calcium and vitamin D demonstrated a 21% increased risk of myocardial infarction (Fig. 1b) with calcium and vitamin D supplementation (RR 95% CI: 1.01, 1.44; *p* = 0.04) with a similar but borderline difference in stroke (*p* = 0.05) and a statistically significant increase for the combination of the two outcomes [RR: 1.16 (95% CI: 1.02, 1.32); *p* = 0.02]. Findings from a patient level data meta-analysis including 24,869 participants from five trials of calcium/ vitamin D supplementation, and the WHI calcium and vitamin D study participants who were not taking personal supplements at baseline, demonstrated an increased risk of myocardial infarction [HR: 1.26 (95% CI: 1.07, 1.47); *p* = 0.005] and stroke [HR: 1.19 (95% CI: 1.02, 1.39); *p* = 0.03] and the combined outcome, but not for death from any cause (*p* = 0.5) [34].

These two meta-analyses and the original study come with a number of concerns, including the heterogeneity of event reporting, that cardiovascular events were not the primary outcome of any included study, that the majority of findings demonstrate weak associations at best, that potentially beneficial effects of similar magnitude have not been emphasised, and that the issue of correction for multiple testing in the secondary analyses has also not been adequately addressed. The lack of dose–response in relation to baseline intake in the WHI study, and the suggestion of opposing effects of baseline dietary intake in the first meta-analysis and personal supplementary intake in WHI are also troubling, as it is difficult to envisage a biological mechanism whereby such a difference could occur.

**Fig. 2:**
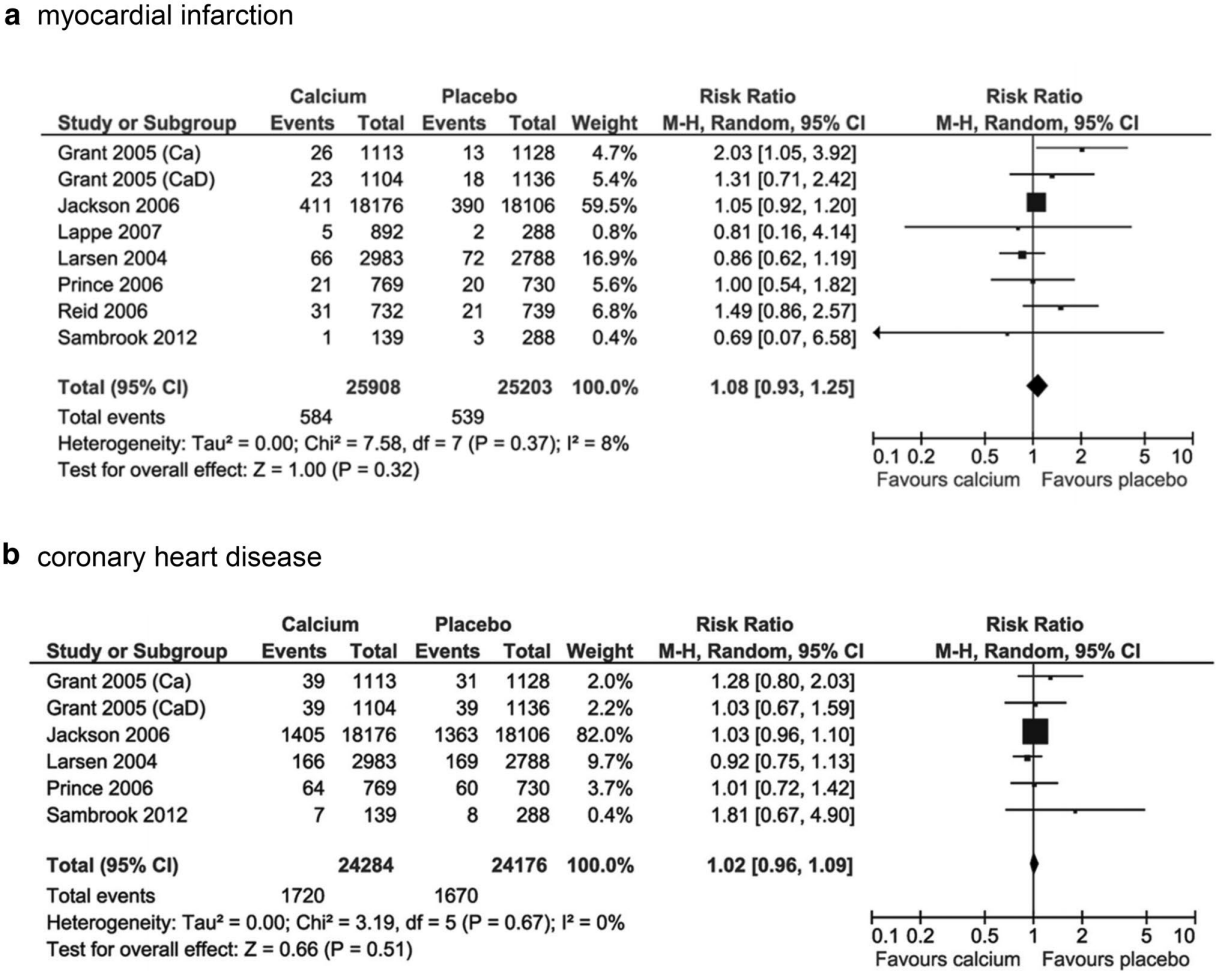
Calcium supplementation with or without vitamin D supplementation and risk of (**a**) myocardial infarction and (**b**) coronary heart disease: random-effects meta-analysis of validated outcomes in women. Reproduced with permission from Lewis et al., 2015 [35]. (**a**) myocardial infarction (**b**) coronary heart disease

Lewis et al. recently undertook a similar meta-analysis to those of Bolland et al., combining trial level data on 63,563 female participants, obtained from published and unpublished results [35], summarised in Fig. 2. The authors focused on women as there are insufficient men in the original trials to form valid conclusions, and the greater rates of cardiovascular events in men than women may lead to erroneous conclusions if randomisation to calcium/placebo is not balanced by sex. The authors also used only trials in which coronary heart disease endpoints were validated and used ICD-based definitions which are globally recognised and encompass different components of coronary heart disease. Given the risk of gastrointestinal side effects with calcium supplements, and that these may be misclassified as cardiovascular events (see above) [31], such adjudication is essential. Furthermore, previous assessments of the validity of self-reported cardiovascular events have demonstrated that confirmation may only be achieved in 60–70% of cases [36, 37]. Bolland et al. also undertook an analysis excluding self-reported outcomes (i.e. limiting the analysis to validated myocardial infarction [38]. Although these results were similar to those from the 2010 meta-analysis (in which 23% of events were ascertained by self-report) [32], it is unclear in this secondary analysis, published in a review article, as to exactly which trials were included in which analysis. In the Lewis study, overall there was no effect of calcium/calcium and vitamin D supplementation on myocardial infarction [RR: 1.08 (95% CI: 0.93, 1.25)], angina pectoris/acute coronary syndrome [RR: 1.09 (95% CI: 0.95, 1.24)] or chronic coronary heart disease [RR: 0.92 (95% CI: 0.73, 1.15)]. In sensitivity analyses, the investigators observed no relationship between supplementation with calcium/ calcium and vitamin D and coronary heart disease or all-cause mortality. However, supplementation with calcium alone was weakly positively associated with myocardial infarction [RR: 1.37 (95% CI: 0.98, 1.92), *p* = 0.07], but it should be noted that the analysis was based on 139 myocardial infarctions in 6333 participants compared with estimates of the effect of calcium with vitamin D based on 1006 events in 45,796 participants. This meta-analysis, unlike the Bolland meta-analyses, included a cluster randomised trial by Larsen et al. [39] However, further sensitivity analyses demonstrated no difference in the findings when cluster randomised trials were excluded. Whilst the authors did not specifically test for an interaction with personal calcium and vitamin D supplement use in the WHI study, a sensitivity analysis in which the WHI participants using personal supplementation at baseline were excluded yielded very similar results overall. This meta-analysis, therefore, does not support the finding from Bolland et al. of a specific effect within those not taking personal calcium and vitamin D supplementation. Whilst the findings of the Lewis et al. meta-analysis are largely reassuring, the weak association for calcium supplementation alone for myocardial infarction should be noted, albeit based on a subgroup with a relatively small number of events, and no consistent effect on coronary heart disease or mortality. Furthermore, none of the meta-analyses have been able to address the associations in men, due to the small number of males in the constituent trials.

The WHI study has been analysed by its own investigators who, in an initial follow-up 8 years from randomisation found no increased risk of myocardial infarction/coronary death or stroke with hazard ratios near one [40]. A comprehensive subgroup analysis demonstrated no increases in cardiovascular events with supplementation [30]. Importantly in this study by Prentice et al., the analysis was stratified by use of personal supplementation. There was no increased risk of myocardial infarction with calcium and vitamin D supplementation in either the whole trial population [HR: 1.03 (95% CI: 0.90, 1.19)], in those who took personal supplements [HR: 0.97 (95% CI: 0.80, 1.17)] or in those who took no personal supplementation [HR: 1.11 (95% CI: 0.90, 1.37)], and no statistically significant difference in the hazard ratios between the strata. Interestingly, the risk of myocardial infarction, coronary heart disease and other outcomes was analysed by time from randomisation, with no evidence of any statistically significant change in the hazard ratio with increasing follow-up time. Although the hazard ratios for myocardial infarction within the first 2 years after randomisation were greater than unity within all participants [HR: 1.19 (95% CI: 0.89, 1.59)] and amongst those who did not take personal supplements [HR: 1.30 (95% CI: 0.86, 1.97)], these were not statistically significant, and over years 2–5, the hazard ratios were close to unity [all participants HR: 0.97 (95% CI: 0.78, 1.21); no personal supplements, HR 1.04 (95% CI: 0.74, 1.47)], with a similar null relationships at > 5 years follow-up. Although clearly limited by variable adherence to medication over the study period, this absence of any time relationship (if anything, there was a decreasing risk with time) with myocardial infarction or other coronary outcomes seems incompatible with any biological mechanism which requires increase in coronary atherosclerosis related to raised calcium concentrations. A further follow-up at up to 5 years after cessation of trial medication again provided reassuring results, finding no difference in coronary heart disease endpoints over 15 years of follow-up in 29,862 women [41], although this was not stratified by use of personal supplements.

**Fig. 3:**
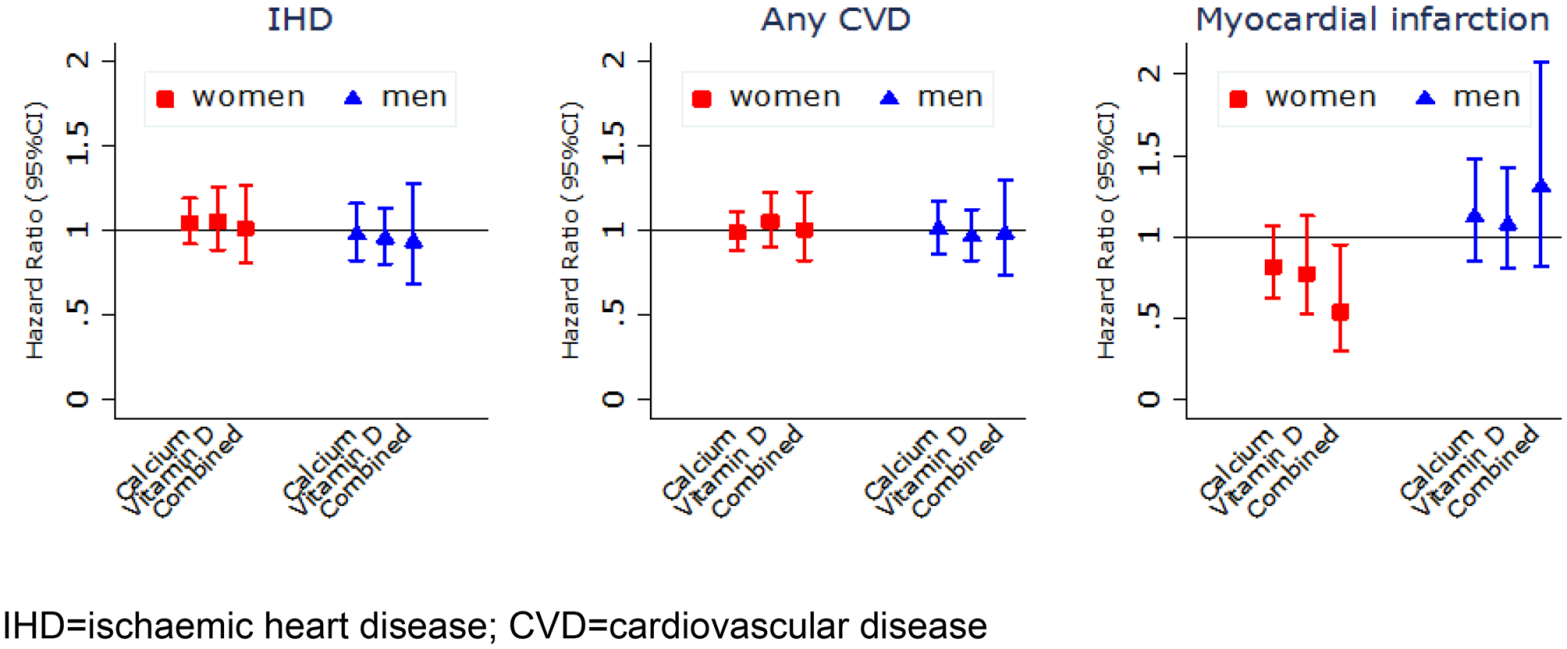
Calcium and/or vitamin D supplementation and cardiovascular outcomes in UK Biobank. Reproduced with permission from Harvey et al., 2018 [46]. *IHD* ischaemic heart disease, *CVD* cardiovascular disease

Several other meta-analyses have been undertaken, but since the underlying evidence base is essentially very similar in all such studies, they remain very much variations upon a theme [42–45]. A limitation of the randomised trial evidence is the low number of men contributing to the trial populations. However, the largest observational study to date comprising over 500,000 men and women in the UK Biobank cohort (Fig. 3), again demonstrated no association between either calcium supplementation, vitamin D supplementation or both with incident ischaemic heart disease, cardiovascular disease myocardial infarction or cardiovascular death [46].

## Calcium (and vitamin D) supplementation and risk of death

**Fig. 4:**
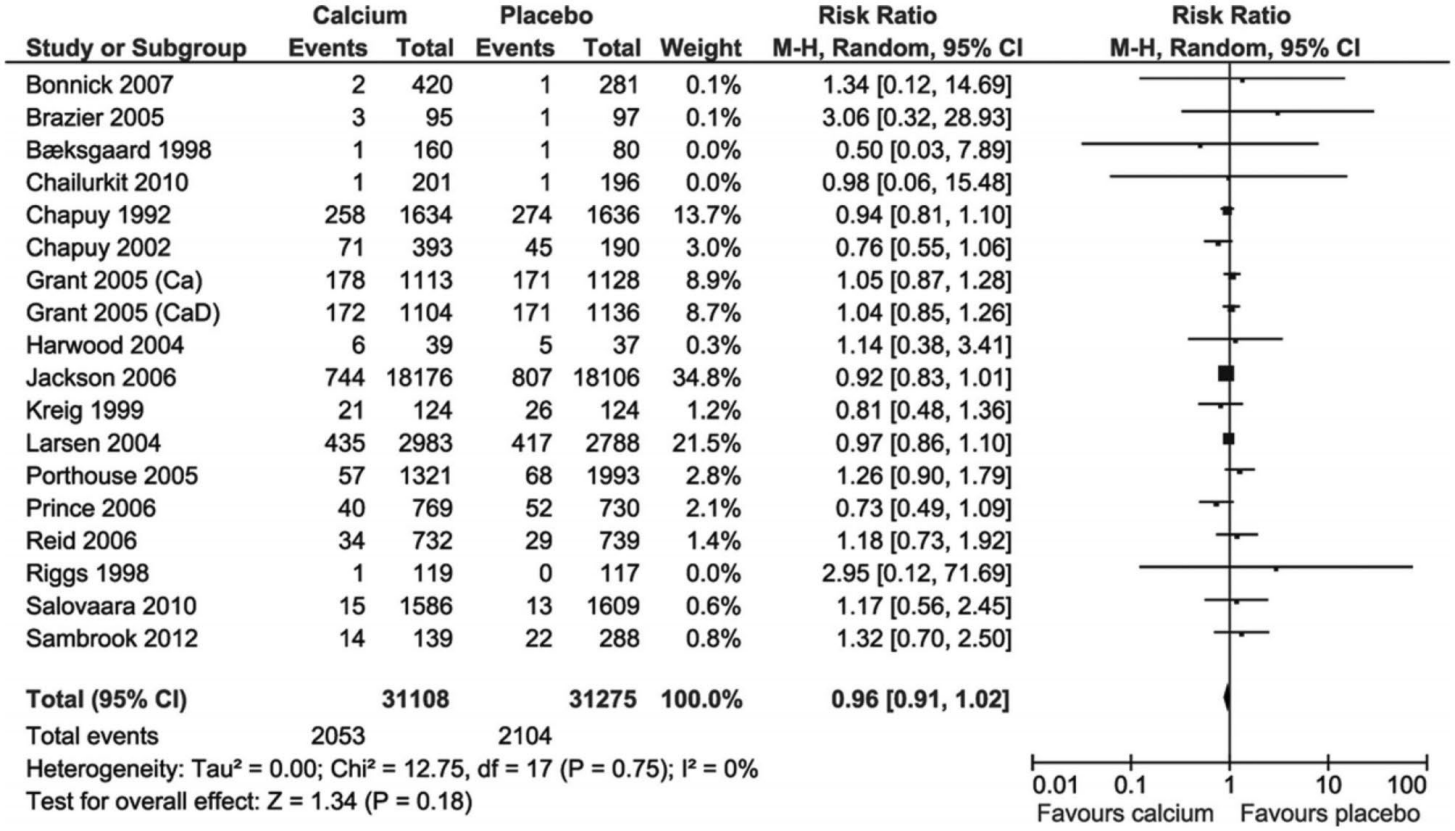
Calcium/vitamin D is supplementation and all-cause mortality compared with no supplementation. Reproduced with permission from Lewis et al., 2015 [35]

It would seem intuitively reasonable that if an intervention leads to an increased risk of a potentially fatal event such as myocardial infarction, then over a reasonable period of time in a large cohort, it would also be associated with an increased risk of death from that cause. Interestingly, to our knowledge, no study has yet demonstrated such an association. Whilst the first meta-analysis by Bolland et al. [32] suggested a possible 7% increase in mortality with calcium supplementation (RR 95% CI: 0.95, 1.19), in their WHI analysis [34], the RR amongst the population not using personal calcium/ vitamin D supplements was very close to unity [RR: 0.99 (95% CI: 0.86, 1.14)]. Amongst those using personal calcium supplements, calcium and vitamin D supplementation were associated with a reduced risk of death [RR: 0.84 (95% CI: 0.73, 0.97)]. In their meta-analysis using verified outcomes, Lewis et al. demonstrated no effect of calcium supplementation on mortality (Fig. 4) [35], a finding similar to that of the DIPART investigators [47], who demonstrated no difference in mortality in an individual patient data meta-analysis of eight studies including RECORD and WHI, and a trial level analysis including a further 16 studies. Again, data from the Women’s Health Initiative demonstrated reassuring findings with mortality somewhat lower in the calcium and vitamin D group compared with placebo [HR: 0.91 (95% CI: 0.83, 1.01)] and this result was similar when examined in those below or above 70 years old. Follow-up of the RECORD study of 5292 participants over 70 years old, who had previously experienced a low trauma fracture [48], demonstrated no effect of calcium supplementation on mortality in an intention-to-treat analysis [all-cause mortality HR: 1.03 (95% CI: 0.94, 1.13); vascular disease mortality HR: 1.07 (95% CI: 0.92, 1.24)]. In a secondary analysis adjusting for treatment received (thus with a reduced number of participants), calcium supplementation again was not statistically significantly associated with all-cause mortality or vascular death, although the hazard ratios were greater than in the ITT analysis [all-cause mortality HR: 1.21 (95% CI: 0.83, 2.05); vascular death HR: 1.43 (95% CI: 0.75, 7.61)]. The trial, therefore, does not provide support either way [48]. Given that the increased risk of myocardial infarction associated with calcium supplementation in the Bolland meta-analyses is relatively modest, and that not all myocardial infarctions result in death, it is possible that existing studies are simply not large enough to detect an effect on mortality [38].

## Mechanistic considerations

### Calcium, phosphate and coronary artery calcification

The mechanisms by which calcium supplementation might increase cardiovascular risk have been much debated. It is notable that in the meta-analyses discussed above, there was no evidence for a dose effect or a convincing temporal relationship between calcium supplementation and cardiovascular disease, both key considerations in establishing a causal relationship [49]. A key argument proposed in favour of a mechanistic link is that the transient rise in serum calcium concentrations consequent to ingestion of a calcium supplement might lead to increased calcium deposition within already damaged coronary artery walls [50]. Indeed, calcification is a recognised component of atherosclerotic disease. However, the increase in serum calcium resulting from supplement ingestion is transient and of modest magnitude, and well below concentrations which would lead to CaHPO_4_ precipitation; at physiological pH and pCO_2_ the serum is only half saturated [51]. Whilst there is, to our knowledge, no direct evidence that such transient increases in calcium concentration cause greater coronary calcification or ischaemic cardiac events, there is evidence from observational studies that, at the population level, serum calcium and/or phosphorus concentrations are positively related to risk of ischaemic cardiac events [52–59]. These studies clearly differ markedly in predictor (calcium × phosphate product, total calcium, corrected calcium, serum phosphorus), outcome (coronary artery calcification, clinical event) and study design (cross-sectional, prospective database); the possibility of confounding and/ or reverse causation remain important considerations in these as in earlier investigations. Furthermore, daily recombinant parathyroid hormone injection, as therapy for osteoporosis, leads to a transient rise in serum calcium concentrations with each dose [60], but the randomised trial evidence, observational studies and post-marketing surveillance data have not demonstrated any concerning signals related to myocardial infarction or other ischaemic cardiac events [61, 62]. Patients with mild hyperparathyroidism (who have a much higher serum calcium concentration than would result from taking calcium supplements) do not have an increased risk of soft tissue calcification, and their risk of MI has not been shown to be increased after adjustment for known CVD risk factors [63]. Finally, in a subset of the WHI participants who underwent computed tomography examination of coronary artery calcification at a mean of 7 years follow-up (754 women aged 50–59 years at baseline), there was no difference in coronary artery calcification score according to calcium and vitamin D treatment versus placebo [64].

### Physiology in health and severe chronic kidney disease

Importantly, the primary abnormality in the development of atherosclerosis is thought to be pathological intimal thickening, with atherosclerotic plaques forming at sites of endothelial damage, rather than exposure to circulating calcium. Indeed, calcification of plaques appears to be related to macrophage apoptosis leading to microcalcifications, which may coalesce [65]. If there were a causal link between calcium supplementation and atherosclerosis then, it could potential be via other cardiovascular risk factors, but there is little evidence for this. Where there have been associations, such as with blood pressure and lipid profile, these have generally been protective [66–70]. Furthermore, whilst there is evidence that calcium supplementation (in phosphate binders) is associated with increased risk of myocardial infarction and death in end-stage renal failure, it is important to appreciate that chronic renal failure leads to a highly perturbed metabolic milieu, in which endothelial dysfunction is an important component [71]. To illustrate, in a study comparing arterial wall calcification in vessels from healthy and chronic renal failure patients, exposure to raised calcium concentrations had no effect on arterial wall calcification in the healthy tissue, but led to increased calcification in the vessels of chronic renal failure patients [72]. The degree of renal failure at which calcium supplementation might become problematic has not been defined. This is an important question, because of the large number of elderly individuals who have mild to moderate renal impairment. The question of whether cardiovascular risk might be raised by calcium supplements specifically in those with pre-existing ischaemic cardiovascular disease remains unanswered. Further work is clearly needed to investigate these mechanistic aspects, but whilst the evidence to date suggests calcium supplementation in end-stage renal failure increases cardiovascular risk, there is no direct evidence of a causal link in healthy individuals.

### Mechanistic inference from causal analyses

Recent investigations have made use of advances in genetic understanding and the widespread availability of genome-wide genotyping in large cohorts, to undertake causal analyses, exploring associations between genetically determined calcium concentrations and cardiovascular outcomes. Among a sample of 184,305 individuals (60,801 coronary artery disease cases [approximately 70% with myocardial infarction] and 123,504 controls), 6 SNPs were estimated to explain about 0.8% of the variation in serum calcium levels [73]. Using an inverse-variance weighted meta-analysis (combining the estimates of the 6 SNPs), the odds ratios per 0.5 mg/dL increase in genetically predicted serum calcium levels were 1.25 (95% CI: 1.08, 1.45) for coronary artery disease and 1.24 (95% CI: 1.05, 1.46) for myocardial infarction. However, in a study of published genome-wide data, amongst 6504 all-cause heart failure cases and 387,652 non-cases of European ancestry, there was no evidence of an association between genetically determined calcium concentrations (7 SNPs) and heart failure [74]. A similar Mendelian randomisation analysis demonstrated no association between genetically determined calcium concentration and any ischaemic stroke subtype in the MEGASTROKE consortium comprising 34,217 cases and 404,630 controls [75]. Again, no convincing association was demonstrated between genetically determined calcium concentration and atrial fibrillation in a further Mendelian randomisation study [76].

Whilst such causal analyses provide weak evidence for a causal association with myocardial infarction, there are substantial limitations with regard to how they should be incorporated into the interpretation of the evidence base as a whole. There are key assumptions which must be satisfied in a Mendelian randomisation analysis. These may be unprovable, such as that the genetic instrument only acts on the outcome through the exposure of interest. Furthermore, given the very small amount of the variance of the exposure explained by the genetic instrument, large cohorts are required to attain statistical power, and the population structure of the cohort can substantially influence the findings [77]. Finally, such studies demonstrate associations between an outcome and lifelong genetically determined exposures. The relevance of this lifelong exposure to the effect of supplementation, which might lead to regular but very transient modest changes in serum calcium concentration, over a defined period in old age, is completely unknown. The gold standard, therefore, remains evidence from randomised, double-blinded, placebo-controlled interventional studies which have validated cardiovascular outcomes. Recent evidence, presented in abstract form, has suggested no effect of calcium supplementation on aortic calcification (assessed from DXA vertebral fracture assessment images) [78] or high-sensitivity troponin [79] (a sensitive marker of myocardial compromise) in 1460 women aged > 70 years, randomised in a double-blind design to either 1200 mg calcium carbonate per day versus matched placebo and followed for 5 years.

## Magnesium

### Background

Magnesium is the fourth most abundant mineral found in the body, with 60% stored in the skeleton [5]. However, consumption of magnesium is variable throughout the world. In the United States, the prevalence of inadequate magnesium intake for adults is about 64% among males and 67% among females, with greater rates of insufficiency at older ages [80]. In the UK National Diet and Nutrition Survey, data suggest that the reference nutrient intake for magnesium is attained in 91.7% adults aged 50 years or over [81].

### Bone effects

Magnesium deficiency has been linked with adverse skeletal outcomes through several potential mechanisms [82], including altered bone mineralization and parathyroid hormone/1,25(OH)_2_-vitamin D physiology; increased bone loss by promoting pro-inflammatory cytokines stimulating remodeling and osteopenia; and increased endothelial dysfunction [83]. Experimental animal and human studies suggest that magnesium deficiency is associated with reduced osteoclastic and osteoblastic activity, osteopenia, and skeletal fragility [84, 85]. A severely magnesium deficient diet leads to impaired bone growth and exacerbation of loss of bone mass in rats [86–88] and mice [89]. Interestingly, animal studies and data from human pregnancies where high doses of magnesium salts have been used to treat pre-eclampsia, have suggested the potential for adverse effects on bone mineralisation at very high intakes of magnesium [90–92].

### Magnesium, osteoporosis and fracture risk

Although the findings from animal studies and experimental investigations suggest the importance of magnesium in bone health, the evidence for a substantial role of magnesium in the pathogenesis of osteoporosis or as a predictor of fracture risk is rather mixed. A recent meta-analysis identified 11 eligible studies involving 2776 postmenopausal women in which magnesium concentrations have been measured and bone density assessed [82]. Overall, there was evidence of a lower concentration of serum magnesium in women with osteoporosis compared with normal controls, with similar findings for bone mineral density at both the femoral neck and lumbar spine, and when stratified by age, but which differed by geographic location. Thus, differences were observed in European women but not in those from Asia. The findings from this meta-analysis are consistent with an earlier systematic review and meta-analysis demonstrating more marginal associations between magnesium dietary intake and bone mineral density at the hip. Given that circulating concentrations of magnesium are tightly regulated, and may not reflect intake closely, it is difficult to properly compare studies of dietary intake with those of serum concentrations. There are few studies of magnesium concentrations or intake with fracture outcomes. Given the critical role of magnesium in cardiovascular health, it could be hypothesised that perturbations in magnesium physiology might lead to fractures through increased propensity to falling through cardiovascular arrhythmias, in addition to any effect on bone mineral density. In the US Women’s Health Initiative Observational Study, total daily magnesium intake was estimated from baseline food frequency questionnaires, together with intake from supplements [2]. Amongst the 73,684 postmenopausal women, baseline hip BMD was 3% higher and whole body BMD was 2% higher in women who consumed > 422.5 mg compared with < 206.5 mg magnesium per day. However, the incidence and relative risk of hip and total fractures did not differ across fifths of intake. In contrast, risk of forearm or wrist fractures increased with higher magnesium intake [multivariate-adjusted hazard ratios: 1.15 (95% CI: 1.01, 1.32) and 1.23 (95% CI: 1.07, 1.42) for fifths 4 and 5, respectively, compared with the lowest fifth], with this finding potentially explained by greater physical activity and exposure to falls risk. In a more recent meta-analysis, fracture outcomes were ascertained from two studies for hip fracture and two for all fractures (both including the WHI study) [3]. The findings provided no evidence for an association between magnesium intake and fracture outcomes, but clearly represent a fairly limited evidence base. With the advent of widespread genome-wide genotypic data on large cohorts, Mendelian randomisation analyses have become possible. A recent such study across various cohorts used a genetic instrument for magnesium concentration consisting of 5 single nucleotide polymorphisms in a sample of 15,366 individuals, and then tested the association in a cohort of 508,253 osteoporotic fracture patients and 53,236 participants from the general population (all of European ancestry) with osteoporosis status documented [93]. The finding that an increase in genetically predicted magnesium concentration of 0.16 mmol/L was associated with a 0.10 g/cm^2^ greater BMD suggests a causal association between magnesium concentration and BMD. However, as described above, the approach relies on several assumptions, which may not be testable, and importantly represents a lifelong genetic influence, and indeed a very small proportion of the variance in overall magnesium concentrations, so cannot really be extrapolated to any effect of dietary intake or supplementation in old age.

### Cardiovascular safety of magnesium

Observational and experimental studies have shown that magnesium can exert beneficial effects on the cardiovascular system by enhancing endothelium-dependent vasodilation, improving lipid metabolism, reducing inflammation, and inhibiting platelet function [4, 94, 95]. Indeed hypomagnesemia (e.g. below 0.65 mmol/L) increases risk of cardiac arrest [96]. In two small randomised, controlled, crossover trials restricted dietary magnesium in healthy postmenopausal women to less than half (101–130 mg) the recommended dietary allowance. This led to increased risk of non-malignant cardiac arrhythmias, changes which were reversed by magnesium supplementation [4, 97, 98]. Severe reductions in dietary magnesium intake may also lead to alterations in oxidative metabolism, glucose homeostasis, and electrolyte balance [97–99]. Although marked reductions in magnesium concentrations or intakes produce adverse effects, whether cardiovascular disease (CVD) risk differs across the normal physiologic concentration range of circulating magnesium or dietary magnesium intake is unclear [4]. A 2005 pooled analysis of prospective cohorts found no significant association between dietary magnesium and IHD (RR: 0.87; 95% CI: 0.67, 1.10) [100].

More recent systematic reviews and meta-analyses, together with causal analyses, have consistently suggested a protective effect of greater magnesium intake and serum concentrations for cardiovascular events within the normal population. It is important to appreciate that these findings should not be extrapolated to disease states such as extremes of serum hypo- or hyper- magnesaemia. An analysis of 532,979 participants from 19 studies (11 studies on dietary magnesium intake, 6 studies on serum magnesium concentrations, and two studies on both) included 19,926 CVD events [5]. The pooled relative risks of total CVD events for the highest versus lowest dietary magnesium intake and serum magnesium concentrations were 0.85 (95% CI: 0.78, 0.92) and 0.77 (95% CI: 0.66, 0.87), respectively. A further very similar meta-analysis that same year (reassuringly) reached the same conclusion, but presented the findings perhaps more usefully [4]. Of 2303 articles, 16 studies met the eligibility criteria; these studies comprised 313,041 individuals and 11,995 CVD, 7534 IHD, and 2686 fatal IHD events. Greater circulating magnesium concentration was associated with a 30% lower risk of CVD [RR: 0.70 (95% CI: 0.56, 0.88) per 0.2 mmol/L)] and possible evidence of a weaker association with lower risks of IHD [0.83 (95% CI: 0.75, 1.05)] and fatal IHD [0.61 (95% CI: 0.37, 1.00)]. Consistent with these findings, there were modest associations between greater dietary magnesium (per 200 mg/d increment) and lower risk of IHD [RR: 0.78 (95% CI: 0.67, 0.92)] and to a lesser extent CVD [(RR: 0.89 (95% CI: 0.75, 1.05)]. The association between dietary magnesium and fatal IHD was found to be non-linear, with an inverse association up to a threshold of 250 mg/d [RR 0.73 (95% CI: 0.62, 0.86)]. Similarly, reassuring protective properties for magnesium intake have been demonstrated with regard to cardiovascular mortality, which, amongst 449,748 individuals (10,313 cardiovascular deaths), cardiovascular mortality was 16% lower in women and 8% lower in men in the remaining population compared with the lowest dietary magnesium intake group [6]. Finally, in a Mendelian randomisation study of 60,801 coronary artery disease cases and 123,504 controls, genetically determined serum magnesium concentration (based on six SNPs) was inversely associated with coronary artery disease [101]. Thus, the odds ratio for coronary artery disease was 0.88 (95% CI: 0.78, 0.99) per 0.1 mmol/L increase in genetically predicted serum magnesium levels, consistent with a causal relationship between lower magnesium concentrations and coronary artery disease, albeit with the caveats mentioned above. The same group undertook similar analyses for genetically determined magnesium serum concentration with the outcomes of heart failure (no association) [74], stroke (inverse association with cardioembolic stroke) [75] and atrial fibrillation (inverse association) [76].

Overall, the evidence suggests that at the level of the population, higher magnesium intake and serum concentrations are generally associated with better health, both in terms of cardiovascular outcomes and, less robustly, with bone outcomes. However, there is no convincing evidence that supplementation with magnesium is an efficacious or practicable route to improving bone mineral density or reducing fracture risk. Indeed, evidence for altered mineralisation with high intakes of magnesium in animal models suggests that care would be needed with such an approach to not inadvertently impair bone health. Currently, adherence to generally advised nutritional guidelines for the recommended intake of magnesium appears a sensible approach.

## Strontium ranelate

### Background

Strontium, an element directly below calcium in group 2 of the periodic table, was first discovered in the eighteenth century as a result of lead mining in Scotland [102, 103]. It is present in the human diet in small quantities for example in leafy greens, grains and seafood [104]. Following the observation that strontium was incorporated into the skeleton, it was recognised that strontium, as with calcium and magnesium, could affect myocardial contractility, influence parathyroid hormone secretion and influence uterine contractions [105, 106]. In the normal human diet, 99% of ingested strontium is deposited in bone, which results in replacement by strontium of around 0.035% calcium in the skeleton [104]. Interestingly, excessive strontium substitution may cause defective bone mineralisation, reminiscent of osteomalacia [104, 107, 108]. Isotopes of strontium have been used to treat bone pain patients with metastatic bone cancer and as a tool for imaging bone lesions [109, 110].

### Bone effects

Strontium lactate and strontium chloride have both been studied as potential treatments for osteoporosis in human and animal studies [107, 111–113]. In the most recent incarnation, strontium is combined with ranelic acid as a carrier to form strontium ranelate. It is taken as a single daily oral dose. Its mechanism of action remains a subject of research, but there is evidence that it increases bone strength by altering bone material properties [103]. Administration of strontium ranelate leads to a substantial increase in BMD at the spine and hip, though part of this increase is artefactual, due to incorporation of strontium (which has a greater atomic mass than calcium) into bone. Studies have shown a 36% relative risk reduction in hip fracture over 3 years in osteoporotic patients [10].

### Anti-fracture efficacy

The anti-fracture efficacy of strontium ranelate in postmenopausal osteoporosis derived originally from two large pivotal randomized controlled trials, for which the results were reported in 2004 [9] and 2005 [10], and which formed basis of its initial clinical indication. The Spinal Osteoporosis Therapeutic Intervention (SOTI) trial included 1442 women aged > 50 years with postmenopausal osteoporosis with at least one prevalent vertebral fracture and a femoral neck *T* score of − 2.8 and a lumbar spine *T* score of − 3.5 [9]. Participants were allocated randomly to either 2 g/day strontium ranelate or matched placebo. Vertebral fractures occurred in 20.9% of women in the strontium ranelate group versus 32.8% in the placebo group over the 3 years’ follow-up. In the second trial (Treatment of Peripheral Osteoporosis Trial: TROPOS), 5091 postmenopausal women with osteoporosis were randomised to either 2 g/day strontium ranelate or matched placebo [10]. Here, 11.2% of strontium ranelate patients had at least one osteoporosis-related nonvertebral fracture, and 8.7% at least one major osteoporotic fracture over the 3 years’ follow-up, compared with 12.9 and 10.4% (respectively) participants in the placebo group.

### Cardiovascular safety of strontium ranelate

In 2013, the Pharmacovigilance Risk Assessment Committee (PRAC) of the European Medicines Agency (EMA) noted concerns over cardiac safety emerging from annual periodic safety update reporting (the mechanism whereby manufacturers submit regular safety data to the EMA) and subsequently recommended a reappraisal of the overall benefit-risk ratio of strontium ranelate [11, 114–116]. The pooled analysis in 7572 postmenopausal women (3803 strontium ranelate and 3769 placebo) indicated an increased risk for myocardial infarction (MI) with strontium ranelate, with estimated annual incidences of 5.7 cases per 1000 patient years versus 3.6 cases per 1000 patient years with placebo [11]. The odds ratio for MI was 1.60 (95% CI: 1.07, 2.38) for strontium ranelate versus placebo (incidences of 1.7 versus 1.1%, respectively). Interestingly, among the cases of MI, fatal events were less frequent with strontium ranelate (15.6%) than with placebo (22.5%). Post-marketing surveillance data covering > 3.4 million patient years of treatment from September 2004 to February 2013 did not support an increased risk of MI [11, 116]. Subsequent observational studies with very large populations have similarly not indicated any adverse signal. Thus, a prospective observational cohort study of 12,076 patients on strontium ranelate did not demonstrate increased risk of cardiac events over the 32.0 ± 9.7 months of follow-up [11, 116, 117]. In a nested case-control study of 112,445 women with treated postmenopausal osteoporosis, of whom 6487 were receiving strontium ranelate, the annual incidence rates for first definite MI, MI with hospitalisation and cardiovascular death were 3.24, 6.13, and 14.66 per 1000 patient years, respectively. As expected, in this analysis within the UK Clinical Practice Research Datalink (CPRD), obesity, smoking and cardiovascular treatments were associated with greater risk of cardiac events, but current use or past use of strontium ranelate was not associated with increased risk for first definite myocardial infarction (compared with patients who had never taken strontium ranelate) [OR: 1.05 (95% CI: 0.68, 1.61) and 1.12 (95% CI: 0.79, 1.58), respectively], hospitalisation with myocardial infarction [0.84 (95% CI: 0.54, 1.30) and 1.17 (95% CI: 0.83, 1.66)], or cardiovascular death [0.96 (95% CI: 0.76, 1.21) and 1.16 (95% CI: 0.94, 1.43)] [118]. The most recent study combined data from three multinational multi-database sources to undertake case-control studies nested within a cohort of new users of strontium ranelate or bisphosphonates [119]. Cases of acute myocardial infarction, venous thromboembolism or cardiovascular death were matched with up to ten controls by sex, year of birth, index date and country. The results indicated that there was no apparent excess risk of acute myocardial infarction with current strontium ranelate versus current bisphosphonate use [Odds Ratio: 0.89 (95% CI 0.70, 1.12)] nor with current versus past strontium ranelate use [0.71 (95% CI: 0.56, 0.91)]. There was evidence for an increased risk of venous thromboembolism with current strontium ranelate compared with current bisphosphonate use [1.24 (95% CI: 0.96, 1.61)], and current versus past strontium ranelate use [1.30 (95% CI: 1.04, 1.62)]. Cardiovascular death was more common with current strontium ranelate versus current bisphosphonate use [1.35 (95% CI: 1.02, 1.80)]. However, when current use was compared with past strontium ranelate use (which better controls for confounding by indication) a reduced risk of cardiovascular death was apparent [0.68 (95% CI: 0.48, 0.96)] [119]. Importantly, this study was undertaken after the change in label and specification of cardiovascular disease as a contraindication to strontium and late use. Cessation of therapies during end-of-life care and residual confounding by indication are suggested by the authors to potentially partly explain these apparently discrepant findings [116, 119].

## Summary and conclusions

Whilst calcium, magnesium and strontium have chemical similarities, the evidence base surrounding their links with bone and cardiovascular health is heterogeneous. Thus, calcium combined with vitamin D supplementation leads to a modest reduction in fracture risk but calcium supplementation is associated with a small increase risk of renal stones, and a more marked increase in risk of gastrointestinal symptoms. The purported links between calcium/vitamin D and cardiovascular outcomes are not consistently supported across the literature and indeed not convincingly apparent when validated outcomes are used; a mechanistic explanation in the context of normal renal physiology is still lacking; and no dose response or convincing temporal relationship between supplementation and cardiovascular outcomes have been demonstrated.

Dietary intake and serum concentrations of magnesium have been positively associated with bone mineral density but links to fracture risk are, as yet, not well characterised; reassuringly, greater dietary intake and serum concentrations appear to be generally protective from cardiovascular events.

Strontium is a trace element and is considered in the context of the skeleton mainly in terms of treatment for osteoporosis, in the form of strontium ranelate. Here, efficacy for vertebral and non-verbal fractures has been demonstrated, but with convincing evidence of increased risk of thromboembolic disease. However, pooled analysis of randomised trial data suggesting an increased risk of myocardial infarction have not been supported by post-marketing surveillance data or analyses of large real-world population datasets.

In conclusion, we suggest that calcium supplementation is most appropriately used in combination with vitamin D supplementation and targeted at those who are deficient in these nutrients, or in combination with antiosteoporosis medications [120]. There is little evidence to support routine magnesium supplementation for bone health. Strontium ranelate, which is now available again as a generic medication, provides a useful therapeutic option for osteoporosis in those at high fracture risk who do not have cardiovascular risk factors.
